# LINC01446/miR-338-3p/APEX1 Axis Promotes Ferroptosis Defense and Progression in Esophageal Squamous Cell Carcinoma

**DOI:** 10.3390/cancers18152496

**Published:** 2026-08-04

**Authors:** Yunlong Jia, Jiaxin Si, Zhendong Zhang, Tianxu Liu, Shuman Zhen, Yan Zhao, Yu Wang, Xuexiao Wang, Jiali Wang, Lihua Liu

**Affiliations:** 1 Department of Tumor Immunotherapy, The Fourth Hospital of Hebei Medical University, Shijiazhuang 050035, China; jyl0314@hebmu.edu.cn (Y.J.); 23032100216@stu.hebmu.edu.cn (J.S.); 25031100169@stu.hebmu.edu.cn (Z.Z.); 25031100171@stu.hebmu.edu.cn (T.L.); 25033100543@stu.hebmu.edu.cn (Y.W.); 48904642@hebmu.edu.cn (X.W.); 49002023@hebmu.edu.cn (J.W.); 2 Department of Tumor Radiotherapy, The Fourth Hospital of Hebei Medical University, Shijiazhuang 050011, China; shumanzhen@hebmu.edu.cn; 3 Department of Medical Oncology, The Fourth Hospital of Hebei Medical University, Shijiazhuang 050011, China; yanzhao0928@hebmu.edu.cn; 4 Cancer Research Institute of Hebei Province, Shijiazhuang 050011, China; 5 China International Cooperation Laboratory of Stem Cell Research, Hebei Medical University, Shijiazhuang 050011, China

**Keywords:** esophageal squamous cell carcinoma (ESCC), LINC01446, apurinic/apyrimidinic endodeoxyribonuclease 1 (APEX1), ferroptosis

## Abstract

Background: Esophageal squamous cell carcinoma (ESCC) is an aggressive malignancy with a poor prognosis, highlighting the urgent need to elucidate its molecular mechanisms to develop targeted therapies. Long non-coding RNAs (lncRNAs) play a critical role in cancer progression. However, the majority of lncRNAs involved in ESCC progression remain to be elucidated. Methods: We integrated bioinformatic analyses of the TCGA and GEO datasets to identify differentially expressed lncRNAs in ESCC, and the biological functions of the candidate lncRNA LINC01446 were investigated using loss-of-function assays in ESCC cell lines, including colony formation, wound healing, Transwell invasion, C11-BODIPY staining, malondialdehyde (MDA) quantification, and glutathione (GSH) evaluation. A nude mouse xenograft model was established for in vivo validation, and the underlying molecular mechanism was explored through RNA sequencing, fluorescence in situ hybridization (FISH), dual-luciferase reporter assays, AGO2-RIP, and rescue experiments. Results: LINC01446 was significantly upregulated in ESCC tissues and cell lines. Based on univariate analysis, high expression of LINC01446 was associated with poorer overall survival (OS). Functional studies showed that LINC01446 knockdown suppressed ESCC cell proliferation, migration, and invasion, promoted ferroptosis-related changes in vitro and was associated with increased lipid peroxidation and possible ferroptosis-related changes in vivo. Mechanistic analyses supported a regulatory relationship in which LINC01446 may function as a competing endogenous RNA (ceRNA) for miR-338-3p, thereby contributing to the upregulation of its downstream target, apurinic/apyrimidinic endodeoxyribonuclease 1 (APEX1), in ESCC cells. Moreover, APEX1 was found to be overexpressed in ESCC and associated with poor OS. Conclusions: This study suggests that the LINC01446/miR-338-3p/APEX1 regulatory axis may contribute to ESCC progression and ferroptosis-related regulation. Additionally, LINC01446 and APEX1 represent promising prognostic biomarkers and therapeutic targets for ESCC.

## Simple Summary

Esophageal squamous cell carcinoma (ESCC) is an aggressive malignancy with a poor prognosis, highlighting the urgent need to elucidate its molecular mechanisms to develop targeted therapies. Long non-coding RNAs (lncRNAs) play a critical role in cancer progression. However, the majority of lncRNAs involved in ESCC progression remain to be elucidated. This study aimed to investigate the expression and role of LINC01446 in ESCC, particularly its involvement in tumor progression and ferroptosis defense. Based on bioinformatics analysis of the TCGA and GEO datasets, LINC01446 was significantly upregulated in ESCC and correlated with poorer overall survival (OS). Functional assays revealed that LINC01446 knockdown suppressed ESCC cell proliferation, migration, and invasion. In addition, its knockdown induced ferroptosis in ESCC cells, as evidenced by elevated oxidized C11-BODIPY staining, increased malondialdehyde (MDA) levels, and decreased glutathione (GSH) levels. Mechanistically, apurinic/apyrimidinic endodeoxyribonuclease 1 (APEX1) was identified as a downstream target of LINC01446 using RNA sequencing and Western blotting, the expression of which was positively correlated in ESCC tissues. Further experiments suggested that cytoplasmic LINC01446 might act as a competing endogenous RNA (ceRNA) that sponges miR-338-3p, thereby alleviating its repressive effect on APEX1 expression. Dual-luciferase and AGO2-RIP assays supported a regulatory relationship among LINC01446, miR-338-3p, and APEX1, consistent with a ceRNA-mediated mechanism. Moreover, LINC01446 knockdown suppressed tumor growth and reduced APEX1 expression in vivo, accompanied by increased MDA levels, suggesting enhanced lipid peroxidation and a possible increase in ferroptosis. These findings suggest that the LINC01446/miR-338-3p/APEX1 axis may contribute to ESCC progression and ferroptosis defense, warranting further investigation into its potential therapeutic and prognostic value.

## 1. Introduction

Esophageal cancer is among the most common malignancies worldwide and poses a substantial global health burden, and in China, esophageal squamous cell carcinoma (ESCC) is the predominant histological subtype and accounts for over 90% of esophageal cancer cases [1]. ESCC is characterized by insidious onset, with a large proportion of patients diagnosed at an advanced stage, which diminishes the opportunity for curative surgery. However, effective agents for salvage therapy in advanced ESCC remain scarce, severely hampering further improvements in patient prognosis [2]. Additionally, the high incidence and mortality rates of ESCC present formidable challenges in cancer treatment, highlighting the urgent clinical imperative to identify novel biomarkers and therapeutic targets for this disease. Therefore, this unmet clinical need underscores the critical necessity of elucidating the underlying molecular mechanisms of ESCC.

Recent studies have illuminated the critical roles of long non-coding RNAs (lncRNAs) in various cancers, including their contributions to tumor progression and therapy resistance [3]. Accumulating evidence has confirmed that multiple lncRNAs are implicated in cancer initiation and progression by regulating gene expression at both transcriptional and post-transcriptional levels [4]. One of the most intensively studied classical biological functions of lncRNAs in cancer-related research is their role as competing endogenous RNAs (ceRNAs) that sponge microRNAs (miRNAs), thereby preventing the latter from binding to the 3′-untranslated regions (3′-UTRs) of target mRNAs [5]. For instance, the lncRNA LHX1-DT upregulates PDCD4 by sponging miR-590-5p in renal cell carcinoma (RCC) [6]. Additionally, increasing evidence has shown that lncRNAs play important roles in ESCC progression by ceRNA-mediated regulatory networks and in influencing tumor growth, metastasis, the epithelial–mesenchymal transition (EMT), and chemoresistance [7]. Our previous work revealed that the lncRNA NORAD sponges miR-224-3p to upregulate metadherin (MTDH) and promote progression and cisplatin resistance in ESCC [8]. In addition, we found that SNHG6 promotes the EMT to activate the metastatic potential of ESCC by regulating the miR-26b-5p/ITGB1 axis [9]. Although the functions of some lncRNAs exerting regulatory effects in ESCC have been elucidated, the expression patterns and biological functions of many ESCC-associated lncRNAs remain unclear. Therefore, further investigations are warranted to unravel the biological roles of these lncRNAs in ESCC.

In this study, we aimed to screen key lncRNAs with clinical prognostic value as candidate genes and further investigate their biological functions and underlying mechanisms in ESCC. We identified LINC01446 as a tumor-promoting lncRNA whose expression pattern and biological function in ESCC had not previously been characterized, and demonstrated its prognostic value in predicting the overall survival (OS) of ESCC patients. Mechanistically, LINC01446 promoted proliferation, migration, invasion, and ferroptosis defense in ESCC cells. Moreover, LINC01446 sponged miR-338-3p to upregulate apurinic/apyrimidinic endodeoxyribonuclease 1 (APEX1), thereby facilitating the aforementioned phenotypes. Taken together, the current investigation seeks to fill critical gaps in our understanding of LINC01446’s role and specific mechanisms in ESCC, and these results may provide valuable insights into ESCC treatment options.

## 2. Materials and Methods

### 2.1. Bioinformatic Analysis

RNA sequencing data were downloaded from The Cancer Genome Atlas (TCGA) to screen for differentially expressed genes between ESCC and normal esophageal epithelial tissues. RNA-seq expression data and corresponding clinical information for esophageal cancer were obtained from TCGA-ESCA. Cases with ESCC histology were retained for downstream analyses, whereas esophageal adenocarcinoma samples were excluded. Normal esophageal tissues available in TCGA-ESCA were used as controls. Expression matrices were processed using the normalized values provided by the database/platform. RNA-seq data were analyzed using the DESeq2 package in R. The microarray profile GSE53625 was downloaded from the Gene Expression Omnibus (GEO) data repository to study differentially expressed lncRNAs between cancer and normal tissues of patients with ESCC. For GSE53625, probe annotation was performed based on the corresponding platform annotation file downloaded from the GEO database. The microarray data were analyzed by using the limma package in R. Genes meeting the criteria of |log2FC| > 2 and FDR < 0.05 were identified as differentially expressed genes. The gene dataset used for Gene Set Enrichment Analysis (GSEA) was downloaded from Ferrdb V2, and the relevant information is listed in Table S1 [10].

### 2.2. Cell Culture

Human ESCC cell lines (KYSE510, TE1, KYSE30, KYSE150, and KYSE410) and a normal esophageal epithelial cell line (HET-1A) were purchased from Procell Life Science & Technology (Wuhan, China). The cell lines underwent authentication and mycoplasma testing prior to culture. The cells were cultured in RPMI-1640 medium (Biological Industries, Beit-Haemek, Israel) supplemented with 10% FBS (VivaCell, Denzlingen, Germany), penicillin (100 U/mL), and phytomycin (100 µg/mL) in an incubator with 5% CO_2_ at 37 °C.

### 2.3. RNA Isolation and Quantitative Reverse-Transcription PCR (qRT-PCR) Assay

Total RNA was prepared using TRIzol reagent (Thermo Fisher Scientific, Waltham, MA, USA), followed by cDNA synthesis using the RevertAid First Strand cDNA Synthesis Kit. Quantitative real-time PCR was carried out using Hieff qPCR SYBR Green Master Mix on an ABI Prism 7900-HT system. The relative expression of target genes was analyzed based on the 2^−ΔΔCT^ approach. GAPDH and U6 small nuclear RNA were used as internal controls to calculate the relative expression levels of lncRNAs and miRNAs. The primers were synthesized by Sangon Biotech (Shanghai, China), and their sequences are listed in Table S2. All assays were conducted in three independent biological replicates.

### 2.4. Western Blotting

Total protein was extracted using the Minute Total Protein Extraction Kit for Animal Cultured Cells/Tissues (Invent Biotechnologies, Eden Prairie, MN, USA). Protein concentrations were measured using a BCA protein assay kit (Report Biotechnology, Shijiazhuang, China). Western blotting was performed as described previously [8]. Primary antibodies against APEX1 (10323-1-AP, Proteintech, Wuhan, China) and beta tubulin (10094-1-AP, Proteintech) were used. The membranes were analyzed using an Odyssey infrared imaging system. The relative expression of proteins was normalized to that of the corresponding control. All Western blotting analyses were performed using three independent biological replicates.

### 2.5. Immunofluorescence

A human ESCC tissue microarray was purchased from Shanghai Outdo Biotech (Shanghai, China) and used to analyze the expression and prognostic role of APEX1 status in ESCC. The microarray was dewaxed and rehydrated with xylene and ethanol. For antigen retrieval, the chip was heated in sodium citrate buffer (pH 6.0) in a water bath for 10 min. After blocking with 5% normal goat serum for 30 min, the chip was incubated with an antibody against APEX1 (10323-1-AP, Proteintech) at a dilution of 1:100 overnight at 4 °C, followed by an FITC-labeled secondary antibody. After incubation in the dark at room temperature, the chip was mounted using an antifade mounting medium containing DAPI (Beyotime, Shanghai, China). Fluorescence intensity was quantified using ImageJ software version 1.54k (National Institutes of Health, Bethesda, MD, USA).

### 2.6. Fluorescence In Situ Hybridization (FISH) Assay

The double FISH assay was conducted in ESCC cells as previously described [8], and the slides were mounted using an antifade mounting medium containing DAPI. After 15 min of mounting, the slides were observed using a confocal microscope. FAM-labeled probes specific to LINC01446 and Cy3-labeled probes specific to miR-338-3p were used in this study. The probe sequences were as follows: LINC01446: 5′-TTCTAATTAAAGTTTCTTCTTGAGTCTTCCATGATTTGAAAGTGT-3′; miR-338-3p: 5′-CAACAAAATCACTGATGCTGGA-3′. RNA FISH probes were designed and synthesized by Servicebio (Wuhan, China). For FISH on the commercial ESCC tissue microarray chip, we used an AF532-labeled probe for LINC01446, which was designed and synthesized by Outdo Biotech.

### 2.7. RNA Immunoprecipitation (RIP) Assay

RNA immunoprecipitation (RIP) was performed using a Magna RIP RNA-Binding Protein Immunoprecipitation Kit (Millipore, Billerica, MA, USA) according to the manufacturer’s instructions. Briefly, cell lysates were incubated with magnetic beads conjugated to an anti-Argonaute-2 (AGO2) antibody (Abcam, Cambridge, MA, USA) or a nonspecific mouse/rabbit IgG antibody as a negative control. An aliquot of the total lysate was collected before immunoprecipitation and used as the input control. After immunoprecipitation and RNA purification, the enrichment of the indicated RNAs was determined by qRT-PCR. RIP-qPCR data were normalized to the input RNA and are presented as the percentage of input. The specificity of the interaction was further evaluated by comparing AGO2 immunoprecipitation with the corresponding IgG control. All experiments were independently repeated three times.

### 2.8. Isolation of Nuclear and Cytoplasmic Fractions

Cytosolic and nuclear proteins were extracted from the ESCC cells using a PARIS Kit (Thermo Fisher Scientific) according to the manufacturer’s instructions. GAPDH and U6 served as fractionation markers for the cytoplasm and nucleus, respectively.

### 2.9. Transfection

The miR-338-3p inhibitor and miRNA inhibitor NC were purchased from Thermo Fisher Scientific. The short hairpin RNA (shRNA) targeting LINC01446 and sh-NC were synthesized and packaged into the pLVX-shRNA2-Puro vector by GenePharma. Cells were transfected using the HighGene Transfection Reagent (ABclonal, Wuhan, China) according to the manufacturer’s instructions. After lentivirus infection, stably transfected ESCC cells were selected using 4 mg/mL puromycin for 10 days. The shRNA sequences used to knock down LINC01446 are listed in Table S3.

### 2.10. Colony Formation Assay

Cell proliferation capacity was measured using a colony formation assay. The ESCC cells in the logarithmic growth phase were harvested and digested into a single-cell suspension. After accurate counting, the cells were seeded into 6-well plates at a density of 400–1000 cells per well. The plates were incubated for 1–3 weeks, with the culture medium refreshed regularly. The culture was terminated upon the formation of visible colonies (defined as > 50 cells). The colonies were washed with PBS, fixed with paraformaldehyde, and stained with crystal violet solution. Finally, the colonies were photographed and counted to calculate the plating efficiency. The experiments were independently repeated three times (*n* = 3 biological replicates).

### 2.11. Ferroptosis Assay

Lipid peroxidation in ESCC cells was evaluated using the BODIPY 581/591 C11 probe (ABclonal). Briefly, the cells were stained with 10 μM BODIPY 581/591 C11 at 37 °C for 30 min, followed by washing with PBS. Fluorescence signals were subsequently observed using a confocal microscope. In addition, intracellular malondialdehyde (MDA) content was determined using an MDA detection kit (Beyotime) in accordance with the manufacturer’s protocol. The specific details are described in our previous study [11]. To quantify intracellular glutathione (GSH) levels in ESCC cells, we used an assay kit (Beyotime) according to the manufacturer’s instructions. All experiments were performed using three independent biological replicates.

### 2.12. Wound Healing Assay

To assess the migratory capacity, the ESCC cells were seeded and cultivated in 6-well plates using serum-free RPMI-1640 medium. Subsequently, a linear scrape was made on the monolayer cells to create an artificial wound, and images of the wound were captured at 0 h and 48 h. Width of the scratch was calculated using ImageJ version 1.54k. The migration rate, representing cell migration, was calculated as follows: [(initial scratch width − 48 h scratch width)/initial scratch width] × 100%. Experiments were independently repeated in triplicate.

### 2.13. Transwell Invasion Assay

To detect the invasive capacity of ESCC cells, Transwell chamber inserts (Corning, New York, NY, USA) were coated with 50 μL of 1 mg/mL Matrigel matrix (Solarbio, Beijing, China) in accordance with the manufacturer’s instructions. A total of 5 × 10^4^ ESCC cells suspended in 200 μL of FBS-free medium were seeded into the upper chamber, and 600 μL of medium supplemented with 10% FBS was added to the lower chamber. Following 24 h of incubation at 37 °C in a 5% CO_2_ atmosphere, non-invasive cells on the membrane were removed using a cotton swab, and the invasive cells were fixed with 0.1% crystal violet. The number of invaded cells was calculated using ImageJ software version 1.54k. Experiments were independently repeated three times.

### 2.14. Dual-Luciferase Reporter Assay

Reporter constructs containing either the wild-type or mutant LINC01446 sequence or the APEX1 mRNA 3′-UTR region with predicted miR-338-3p binding sites were designed and synthesized by GenePharma (Shanghai, China). Luciferase activities were determined using the dual-luciferase reporter assay system provided by GenePharma in accordance with the manufacturer’s instructions. Firefly luciferase activity was normalized to the corresponding Renilla luciferase activity to obtain the relative reporter activity. Three independent biological replicates were performed for each assay. All assays were conducted in three independent biological replicates.

### 2.15. Nude Mouse Xenograft Model

BALB/c nude mice aged 4–6 weeks were purchased from HFK Bioscience (Beijing, China). The 10 nude mice were randomly divided into two groups: sh-LINC01446 and sh-NC. A total of 5 × 10^6^ ESCC cells transfected with sh-LINC01446 or sh-NC were subcutaneously injected into the flank of each mouse. The length and width of the tumors were measured every 4 days by the laboratory staff, who were unaware of the animal grouping assignments, and the tumor volume was calculated using the formula: length × width^2^/2. After four weeks of injection, the mice were euthanized by cervical dislocation, and the tumors were harvested for analyses.

### 2.16. Immunohistochemistry (IHC)

IHC staining and subsequent evaluation were carried out following the procedures established in our earlier work [8]. A rabbit polyclonal anti-APEX1 antibody (10323-1-AP, Proteintech) was applied as the primary antibody. For each tissue section, the proportion of positively stained cells and the staining intensity were assessed using the IHC Profiler plugin in ImageJ version 1.54k. The final IHC score was obtained by multiplying the staining intensity score by the score for the percentage of positive cells. Samples with an IHC score of 6 or lower were classified as having low APEX1 expression, whereas those with scores above 6 were defined as having high expression.

### 2.17. Statistical Analysis

Statistical analyses were performed using IBM SPSS Statistics 29.0 (IBM, Armonk, NY, USA). Survival analysis was performed using the Cox proportional hazards model, and curves were drawn using the survival and survminer packages in R. Other statistical graphics were generated using GraphPad Prism 10.2.3 (GraphPad Software, Boston, MA, USA). The graphical abstract was generated using BioRender (agreement number: RO299LOZYR). All measurement data are presented as mean ± SD. Student’s *t*-test was used to analyze the data with equal variance, and the Mann–Whitney U test was used for data without equal variance. The Kaplan–Meier analysis was employed for univariate survival analysis, while the Cox proportional hazards regression model was used for multivariate survival analysis. Statistical significance was set at *p* < 0.05, and all *p* values were two-tailed.

## 3. Results

### 3.1. LINC01446 Is Upregulated in ESCC and Correlated with Poor Prognosis

**Figure 1:**
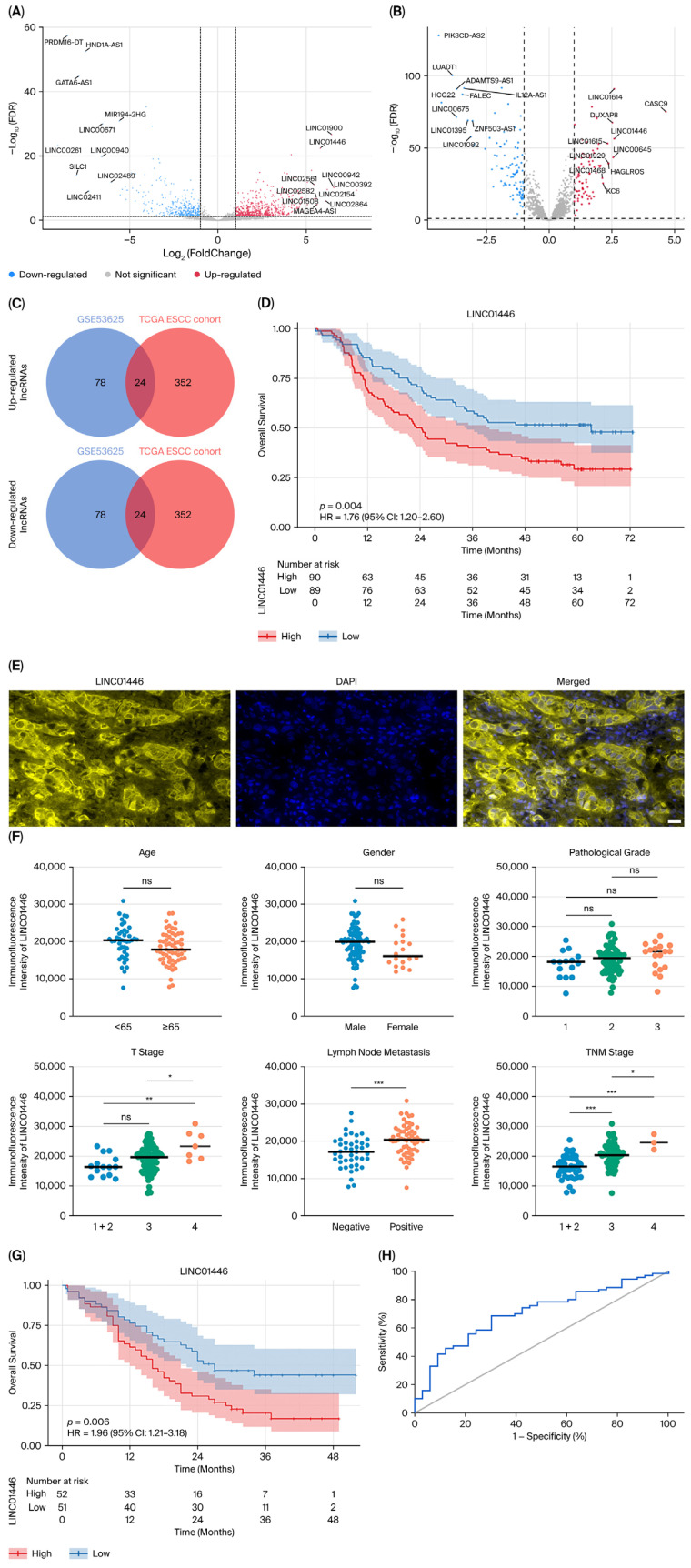
LINC01446 is upregulated in ESCC and is correlated with poor prognosis in patients with ESCC. (**A**) Volcano plot showing the differentially expressed lncRNAs between ESCC and normal esophageal tissues in the ESCC cohort from the TCGA database. The top 20 lncRNAs with the most significant differences in expression levels are labeled. (**B**) Volcano plot showing the differentially expressed lncRNAs between ESCC and normal esophageal tissues in the GSE53625 dataset from the GEO database. (**C**) Overlapping lncRNAs in the GEO GSE53625 dataset and the ESCC cohort in the TCGA database. (**D**) The correlation between LINC01446 expression and the prognosis of patients with ESCC in the GSE53625 dataset was analyzed using Kaplan–Meier analysis. (**E**) Representative images of LINC01446 expression in ESCC tissues, as detected using the FISH. Scale bar = 50 μm. (**F**) Correlation between LINC01446 expression and clinical characteristics of patients with ESCC. (**G**) The correlation between LINC01446 expression and the prognosis of patients with ESCC was analyzed using Kaplan–Meier analysis. (**H**) ROC curves of LINC01446 for predicting the OS of patients with ESCC. ns, *p* > 0.05, * *p* < 0.05, ** *p* < 0.01, *** *p* < 0.001.

First, we analyzed the TCGA ESCC cohort to screen for differentially expressed lncRNAs between ESCC and normal esophageal epithelial tissues. By setting the cut-off values as a fold-change of 2 and FDR < 0.05, we found 969 differentially expressed lncRNAs, among which 593 lncRNAs were upregulated and 376 lncRNAs were downregulated in ESCC tissues compared to normal esophageal squamous epithelial tissues (Figure 1A and Figure S1). We then analyzed GSE53625, a GEO profile that contains paired tumor and normal tissues of 179 ESCC patients, to further confirm the key lncRNAs in ESCC. We found 184 differentially expressed lncRNAs, of which 82 were upregulated and 102 were downregulated in ESCC tissues (Figure 1B and Figure S2). By overlapping the results from the TCGA ESCC cohort and GSE53625, we identified 83 differentially expressed lncRNAs, among which 59 were upregulated and 24 were downregulated in ESCC tissues compared to normal tissues (Figure 1C), and ten lncRNAs with the most significantly upregulated expression were selected as candidates, including LINC00392, LINC00942, LINC01446, LINC01614, LINC00460, LINC00707, LINC01615, POU6F2-AS2, LINC00626, and LINC01592. Next, we determined the prognostic significance of the candidate lncRNAs in ESCC by analyzing patient postoperative survival time in GSE53625. Among the candidate lncRNAs, only LINC01446 was significantly correlated with the OS of ESCC patients, whereas the other candidate lncRNAs did not show a significant correlation (Figure 1D and Figure S3).

To confirm LINC01446 expression at the tissue level, we used a commercial ESCC tissue microarray chip and FISH. As shown in Figure 1E, LINC01446 was localized in the cytoplasm of ESCC tissues. Subsequently, we quantified its expression in ESCC cells and analyzed its correlation with clinical and pathological characteristics, revealing that LINC01446 expression was significantly correlated with invasion range, lymph node metastasis, distant metastasis, and TNM stage but not with age, gender, or pathological grade (Figure 1F). We then conducted a survival analysis to determine its prognostic significance and found that LINC01446 was negatively correlated with the OS of ESCC patients (*p* = 0.006, Figure 1G). To further verify the predictive role of LINC01446 in ESCC, we drew a receiver operating characteristic (ROC) curve and calculated the area under the ROC (AUC), yielding an AUC of 0.7069 in ESCC patients (95% CI: 0.6033–0.8106, *p* < 0.001, Figure 1H), thus suggesting that LINC01446 might be a potential predictive biomarker in ESCC. Additionally, we performed Cox proportional hazards regression analyses to investigate the prognostic significance of these factors in ESCC. Univariate Cox regression analysis was first conducted to identify variables (e.g., gender, age, TNM stage, invasion range, lymph node metastasis, LINC01446 expression, APEX1 expression) for inclusion in the multivariate model. The variables included the TNM stage, lymph node metastasis, pathological grade, and expression levels of LINC01446 and AKR1C1. Among these variables, only lymph node metastasis was significantly associated with OS in patients with ESCC (HR = 1.577, 95% CI: 1.229–2.024, *p* < 0.001). Therefore, although the expression of LINC01446 and APEX1 is closely associated with OS in ESCC patients, they cannot be regarded as independent prognostic factors.

### 3.2. LINC01446 Promotes Proliferation, Migration, Invasion and Ferroptosis Defense by Upregulating APEX1 in ESCC

**Figure 2:**
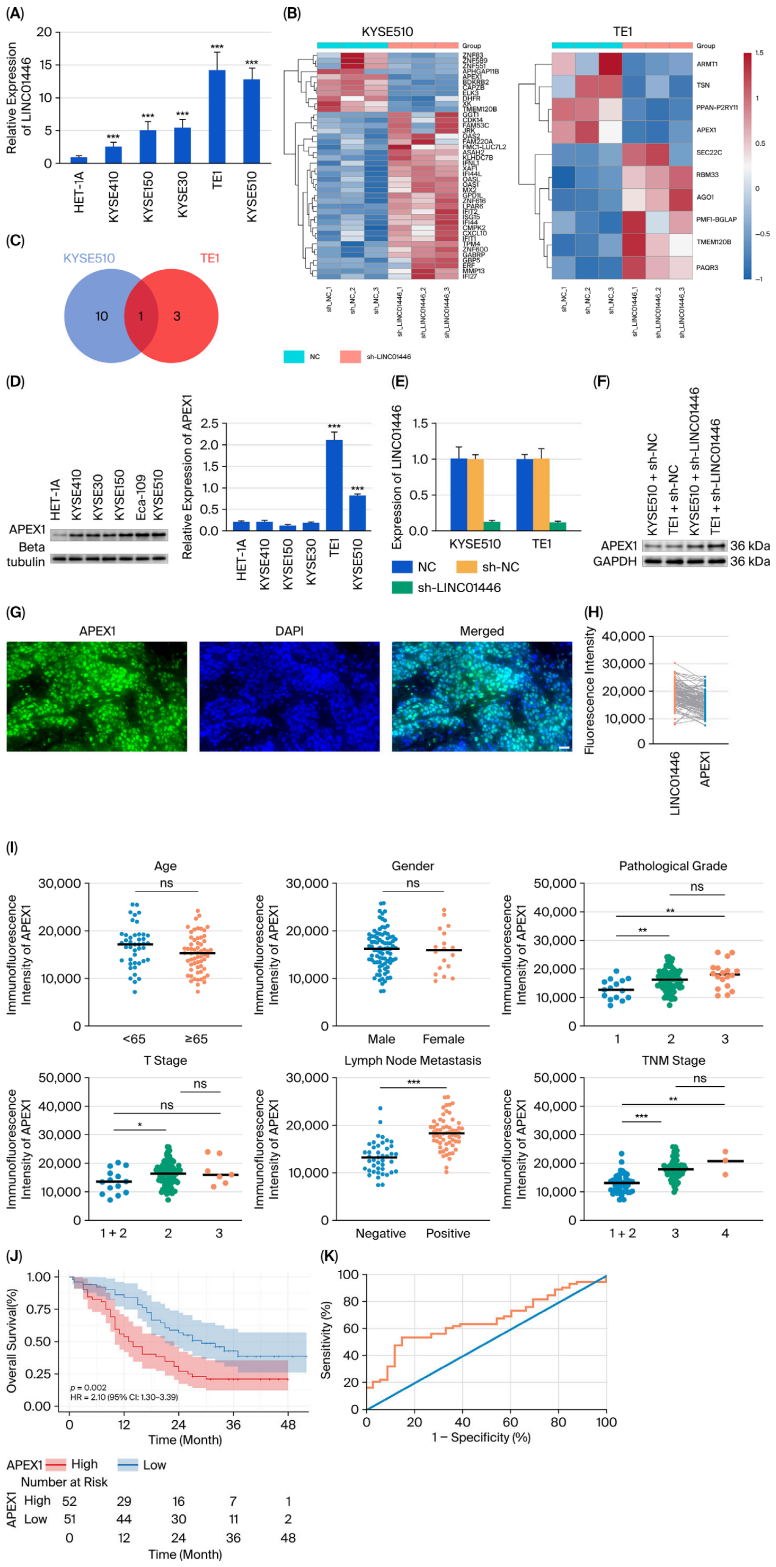
APEX1 is the target gene of LINC01446 in ESCC and is correlated with poor prognosis in patients with ESCC. (**A**) Expression profile of LINC01446 in ESCC cell lines (*n* = 3 biological replicates). (**B**) Protein-coding genes with altered expression levels after LINC01446 knockdown in KYSE510 and TE1 cell lines. (**C**) Overlapping protein-coding genes with altered expression levels after LINC01446 knockdown in KYSE510 and TE1 cell lines. (**D**) Expression of APEX1 in ESCC cell lines (*n* = 3 biological replicates). The expression of APEX1 was analyzed using Student’s *t*-test. (**E**) Knockdown efficiency of sh-LINC01446 in KYSE510 and TE1 cell lines, as detected using qRT-PCR (*n* = 3 biological replicates). The expression of LINC01446 was analyzed using Student’s *t*-test. (**F**) Effects of LINC01446 knockdown on APEX1 expression in KYSE510 and TE1 cell lines, as verified by conducting Western blotting (*n* = 3 biological replicates). The expression of LINC01446 was analyzed using Student’s *t*-test. (**G**) Representative images of APEX1 expression in ESCC tissues, as detected using immunofluorescence staining. Scale bar = 50 μm. (**H**) Correlation between LINC01446 and APEX1 expression in ESCC tissue microarray (*n* = 103). The correlation between LINC01446 and APEX1 was assessed using Pearson’s correlation analysis. (**I**) Correlations between APEX1 expression and clinical characteristics of patients with ESCC, which were analyzed using Student’s *t*-test. (**J**) The correlation between APEX1 expression and the prognosis of patients with ESCC was analyzed using Kaplan–Meier analysis. (**K**) ROC curves of LINC01446 for predicting the OS of patients with ESCC. ns, *p* > 0.05, * *p* < 0.05, ** *p* < 0.01, *** *p* < 0.001.

Next, we examined the expression of LINC01446 in ESCC cell lines using qRT-PCR. The expression of LINC01446 was higher in all ESCC cell lines than in the HET-1A cell line (Figure 2A), and among these ESCC cell lines, KYSE510 and TE1 cells showed the highest expression of LINC01446; therefore, these two cell lines were selected for subsequent experiments. To ascertain the specific target mRNAs of LINC01446 in ESCC, we transfected KYSE510 and TE1 cells with sh-LINC01446 and conducted high-throughput sequencing of their mRNAs, and the results were uploaded to the GEO database under the GSE283632 profile. We identified 42 and 10 differentially expressed protein-coding genes in KYSE510 and TE1 cells, respectively, in the sh-LINC01446 group compared to the corresponding cells in the sh-NC group (Figure 2B). Additionally, among the differentially expressed protein-coding genes, only APEX1 was shared between the two cell lines under the predefined statistical thresholds (Figure 2C). This limited overlap suggests that the transcriptional changes associated with LINC01446 knockdown may be cell context-dependent rather than uniformly conserved across ESCC cell lines. We then performed Western blotting to determine APEX1 expression in ESCC cells, and as shown in Figure 2D, its expression was significantly higher in KYSE510 and TE1 cells than in HET-1A cells. To confirm LINC01446’s regulatory effect on APEX1, we knocked down its expression using shRNA (Figure 2E) and conducted Western blotting in KYSE510 and TE1 cells, yielding significantly reduced APEX1 expression in KYSE510 and TE1 cells (Figure 2F). To confirm its potential regulatory effect on APEX1 at the tissue level, we conducted immunofluorescence on a tissue microarray chip to detect APEX1 expression in ESCC. As shown in Figure 2G, immunofluorescent staining of APEX1 was located in the nucleus of ESCC cells. Subsequently, we quantified APEX1 expression in ESCC cells and analyzed its correlation with LINC01446. We found that LINC01446 was modestly positively correlated with APEX1 expression (Pearson’s r = 0.383, *p* < 0.001, Figure 2H). Additionally, APEX1 expression was significantly correlated with pathological grade, invasion range, lymph node metastasis, distant metastasis, and TNM stage but not with age and gender (Figure 2I). Furthermore, we conducted a survival analysis to determine APEX1’s prognostic significance and found its expression was negatively correlated with the OS of ESCC patients (*p* = 0.002, Figure 2J). Similarly, we drew the ROC curve and calculated the AUC to further verify its predictive role in ESCC, yielding an AUC of 0.6649 for OS in ESCC patients (95% CI: 0.5592–0.7707, *p* = 0.007, Figure 2K), thus suggesting that APEX1 is a potential predictive biomarker for ESCC.

**Figure 3:**
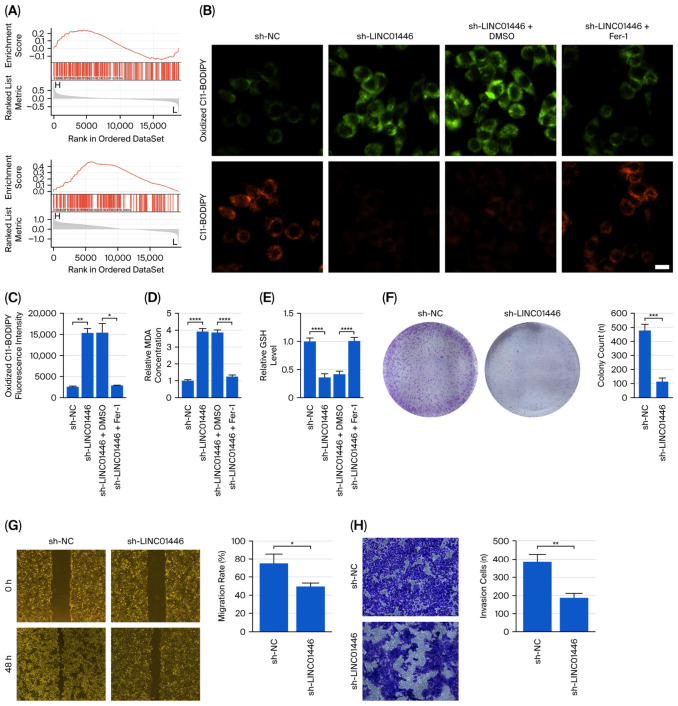
Effect of LINC01446 on phenotypes and ferroptosis in ESCC cells in vitro. (**A**) GSEA of ferroptosis-related enrichment for protein-coding genes with altered expression levels after LINC01446 knockdown in KYSE510 and TE1 cell lines. (**B**) Effect of LINC01446 knockdown on intracellular lipid peroxidation levels in KYSE510 cells, as detected using the C11-BODIPY probe. (**C**) Quantitative analysis of fluorescence signals detected by the C11-BODIPY probe in KYSE510 cells (*n* = 3 biological replicates). The oxidized C11-BODIPY fluorescence intensity was analyzed using Student’s *t*-test. (**D**) Quantitative analysis of MDA, a lipid peroxidation product, in KYSE510 cells after LINC01446 knockdown (*n* = 3 biological replicates). MDA levels were analyzed using Student’s *t*-test. (**E**) Effects of LINC01446 knockdown on GSH levels in KYSE510 cells (*n* = 3 biological replicates). GSH levels were analyzed using Student’s *t*-test. Scale bar = 0.2 μm. (**F**) Effect of LINC01446 knockdown on the proliferation capacity of KYSE510 cells, as evaluated by the colony formation assay (*n* = 3 biological replicates). The counts of colonies were analyzed using Student’s *t*-test. (**G**) Effect of LINC01446 on the migration of KYSE510 cells, as detected using the wound healing assay (*n* = 3 biological replicates). The migration rates were analyzed using Student’s *t*-test. (**H**) Effect of LINC01446 on the invasion of KYSE510 cells, as detected using the Transwell invasion assay (*n* = 3 biological replicates). The numbers of invaded cells were analyzed using Student’s *t*-test. * *p* < 0.05, ** *p* < 0.01, *** *p* < 0.001, **** *p* < 0.0001.

Given that APEX1 is a potential ferroptosis suppressor [12], we next investigated whether it affected ferroptosis in ESCC. Thus, we performed GSEA based on the protein-coding gene expression signature of the GSE283632 profile to reveal APEX1’s role in ferroptosis. The GSEA results showed that APEX1 was positively correlated with ferroptosis suppressors (*p* < 0.001, Figure 3A) but not with ferroptosis drivers (*p* = 0.588, Figure 3A). Ferroptosis-associated alterations were assessed by C11-BODIPY staining, MDA quantification, and GSH measurement to investigate the role of APEX1 in ESCC cells. In KYSE510 and TE1 cells, LINC01446 knockdown resulted in a significant increase in oxidized C11-BODIPY signals and MDA levels, accompanied by a reduction in GSH content (Figure 3B–E and Figure S4A–C). Moreover, the ferroptosis suppressor Ferrostatin-1 (Fer-1) attenuated the effects of LINC01446 knockdown, as evidenced by decreased lipid peroxidation, reduced MDA production, and restoration of GSH levels (Figure 3B–E and Figure S4A–C). APEX1 has been shown to promote the migration and invasion of cancer cells [13,14]. Therefore, we next determined the effect of APEX1 expression on the phenotypes of ESCC cells. LINC01446 knockdown significantly retarded proliferation, migration, and invasion in KYSE510 and TE1 cells (Figure 3F–H and Figure S4D–F). Therefore, these results suggest that LINC01446 may contribute to ferroptosis defense and progression in ESCC.

### 3.3. LINC01446 Sponges miR-338-3p to Upregulate APEX1 in ESCC

**Figure 4:**
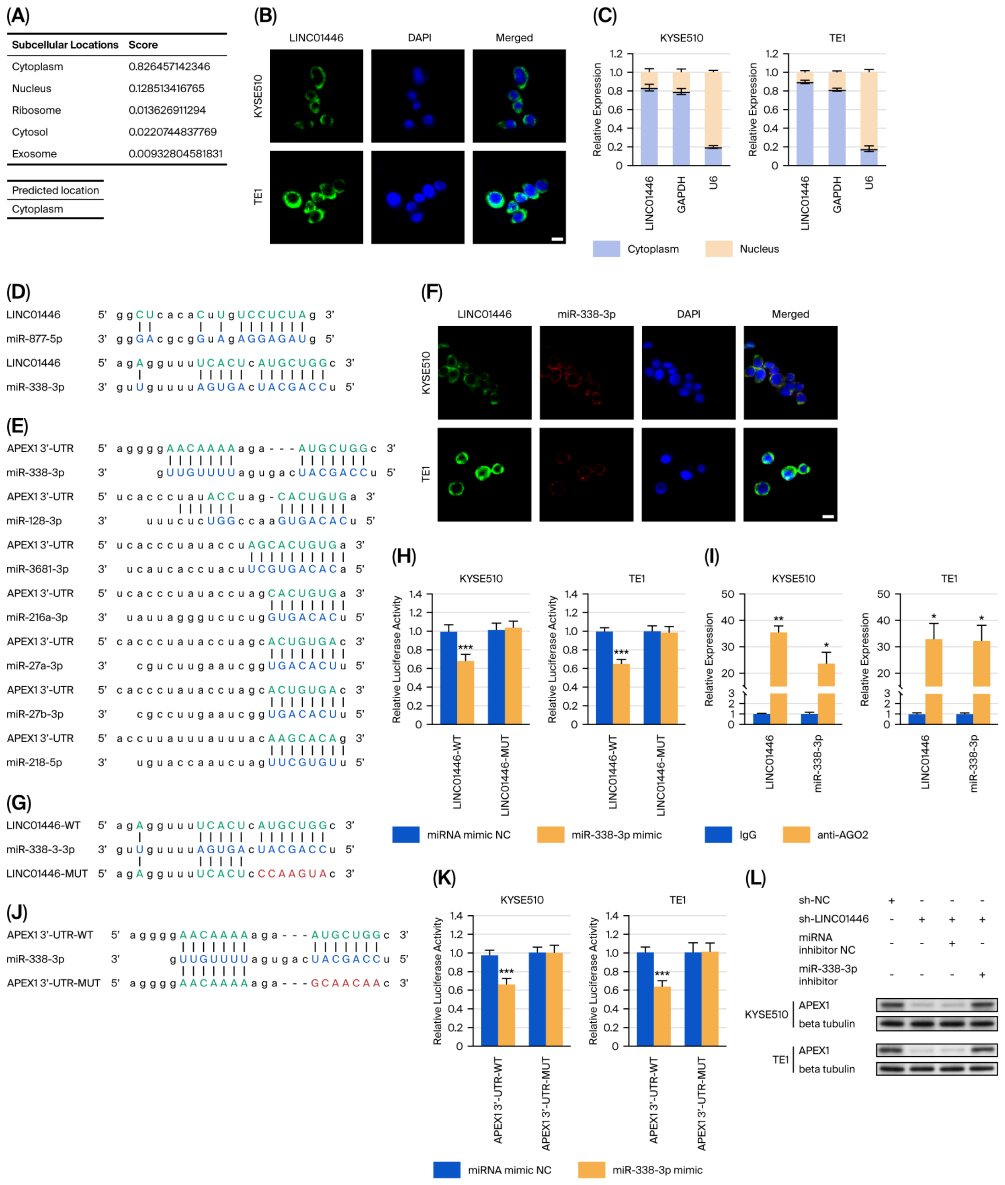
LINC01446 regulates the expression of APEX1 in ESCC by sponging miR-338-3p. (**A**) Subcellular localization of linc01446 was predicted using the online tool lncLocator. (**B**) Representative images of the subcellular localization of LINC01446 in KYSE510 and TE1 cells, as detected using FISH. LINC01446 was labeled green, and the nuclei were stained with DAPI (blue). Scale bar = 20 μm. (**C**) Quantitative analysis of LINC01446 subcellular localization in KYSE510 and TE1 cells, detected using isolation of nuclear and cytoplasmic fractions followed by qRT-PCR (*n* = 3 biological replicates). Relative expression of genes was analyzed using Student’s *t*-test. (**D**) Predicted binding sites of LINC01446 for miR-877-5p and miR-338-3p. (**E**) Binding sites between candidate miRNAs and the 3′-UTR of the APEX1 mRNA. (**F**) Representative images of the subcellular localization of LINC01446 and miR-338-3p in KYSE510 and TE1 cells, as detected using FISH. LINC01446 and miR--338-3p are labeled green and red, respectively, while nuclei are labeled with DAPI (blue). Scale bar = 20 μm. (**G**) Predicted binding sequence between LINC01446 and miR-338-3p, and the corresponding mutant sequence. (**H**) Relative luciferase activity of ESCC cells transfected with LINC01446-WT or LINC01446-MUT, along with miR-338-3p mimic or miRNA mimic NC, as detected using a luciferase reporter assay (*n* = 3 biological replicates). Relative luciferase activities were analyzed using Student’s *t*-test. (**I**) Enrichment level of LINC01446 and miR-338-3p in the AGO2 and IgG pellets of KYSE510 and TE1 cells (*n* = 3 biological replicates). Relative gene expression was analyzed using Student’s *t*-test. (**J**) Predicted binding sequence between the 3′-UTR of APEX1 mRNA and miR-338-3p, and the corresponding mutant sequence. (**K**) A luciferase reporter assay was conducted to measure the relative luciferase activity of ESCC cells transfected with APEX1 3′-UTR-WT or APEX1 3′-UTR-MUT, along with the miR-338-3p mimic or miRNA mimic NC (*n* = 3 biological replicates). Relative luciferase activities were analyzed using Student’s *t*-test. (**L**) Western blotting was performed to validate the LINC01446/miR-338-3p/APEX1 axis in KYSE510 and TE1 cells. * *p* < 0.05, ** *p* < 0.01, *** *p* < 0.001.

Since the function of lncRNAs partly depends on their subcellular localization, we determined the dominant localization of LINC01446 in ESCC cells using FISH. First, we used lncLocator (http://www.csbio.sjtu.edu.cn/bioinf/lncLocator/, accessed on 2 June 2026), an online algorithm developed by Shanghai Jiao Tong University [15], to predict its subcellular location, which was determined to be the cytoplasm (Figure 4A). We then conducted FISH to validate the predicted results and found that LINC01446 was mainly located in the cytoplasm of the ESCC cells (Figure 4B). In addition, we conducted nuclear and cytoplasmic fraction isolation experiments followed by qRT-PCR. The results showed that the cytoplasmic expression levels of LINC01446 were 83.38 ± 3.42% and 89.86 ± 1.73% in KYSE510 and TE1 cells, respectively, further confirming that the cytoplasm is the dominant subcellular location of LINC01446 in ESCC cells (Figure 4C). Some lncRNAs located in the cytoplasm act as ceRNAs that sponge matched miRNAs, thereby neutralizing their suppressive effects on target mRNAs [16]. Thus, we analyzed bioinformatics algorithms to identify the potential miRNAs mediating the regulatory effect of LINC01446 on APEX1. By analyzing Starbase [17], we found that miR-877-5p and miR-338-3p had the potential to interact with LINC01446 (Figure 4D). Then, we used TargetScan, an online bioinformatics algorithm, to predict the miRNAs which could potentially bind with the 3′-UTR of APEX1 mRNA, and seven miRNAs: miR-338-3p, miR-128-3p, miR-3681-3p, miR-216a-3p, miR-27a-3p, miR-27b-3p and miR-218-5p (Figure 4E). Based on the two analyses above, we selected miR-338-3p as the candidate downstream miRNA of LINC01446 for further study and determined the subcellular locations of LINC01446 and miR-338-3p. As shown in Figure 4F, LINC01446 and miR-338-3p were both located in the cytoplasm of KYSE510 and TE1 cells. Taken together, we focused on miR-338-3p to further study LINC01446’s regulatory effect on APEX1.

To confirm the presence of the LINC01446/miR-338-3p/APEX1 axis, we conducted a dual-luciferase reporter assay. First, we constructed luciferase reporters carrying LINC01446 with a mutation in the predicted binding sites for miR-338-3p (Figure 4G). Then, the reporters carrying LINC01446-MUT or LINC01446-wild type (WT) were introduced into KYSE510 and TE1 cells, followed by co-transfection with miR-338-3p mimics. Co-transfection of miR-338-3p mimics significantly decreased luciferase activity in ESCC cells transfected with the reporter carrying LINC01446-WT. In contrast, the miR-338-3p mimic did not decrease luciferase activity in ESCC cells transfected with the reporter carrying LINC01446-MUT (Figure 4H). Next, we conducted AGO2-RIP and qRT-PCR to verify whether lLINC01446 and miR-338-3p occupied the same AGO2 protein to form an RNA-induced silencing complex (RISC). We found that LINC01446 and miR-338-3p were significantly enriched in the AGO2 pellets of KYSE510 and TE1 cells compared to that in IgG pellets (Figure 4I). In addition, we constructed luciferase reporters carrying APEX1 mRNA with a mutation in the predicted binding sites for miR-338-3p (Figure 4J). As shown in Figure 4K, co-transfection with the miR-338-3p mimic significantly reduced luciferase activity in the ESCC cells transfected with the reporter carrying APEX1 3′-UTR-WT; however, it did not reduce luciferase activity in those cells transfected with the APEX1 3′-UTR-MUT reporter. These results suggest that LINC01446 may directly bind to miR-338-3p and form an RISC to regulate APEX1 expression in ESCC. We then performed Western blotting to confirm the presence of the LINC01446/miR-338-3p/APEX1 axis. The results showed that LINC01446 knockdown significantly reduced APEX1 expression in KYSE510 and TE1 cells, while the miR-338-3p inhibitor rescued this downregulation (Figure 4L). In conclusion, these results demonstrate that LINC01446 sponges miR-338-3p to upregulate APEX1 expression in ESCC cells.

### 3.4. LINC01446/miR-338-3p/APEX1 Axis Promotes Migration and Invasion, and Suppresses Ferroptosis, in ESCC

**Figure 5:**
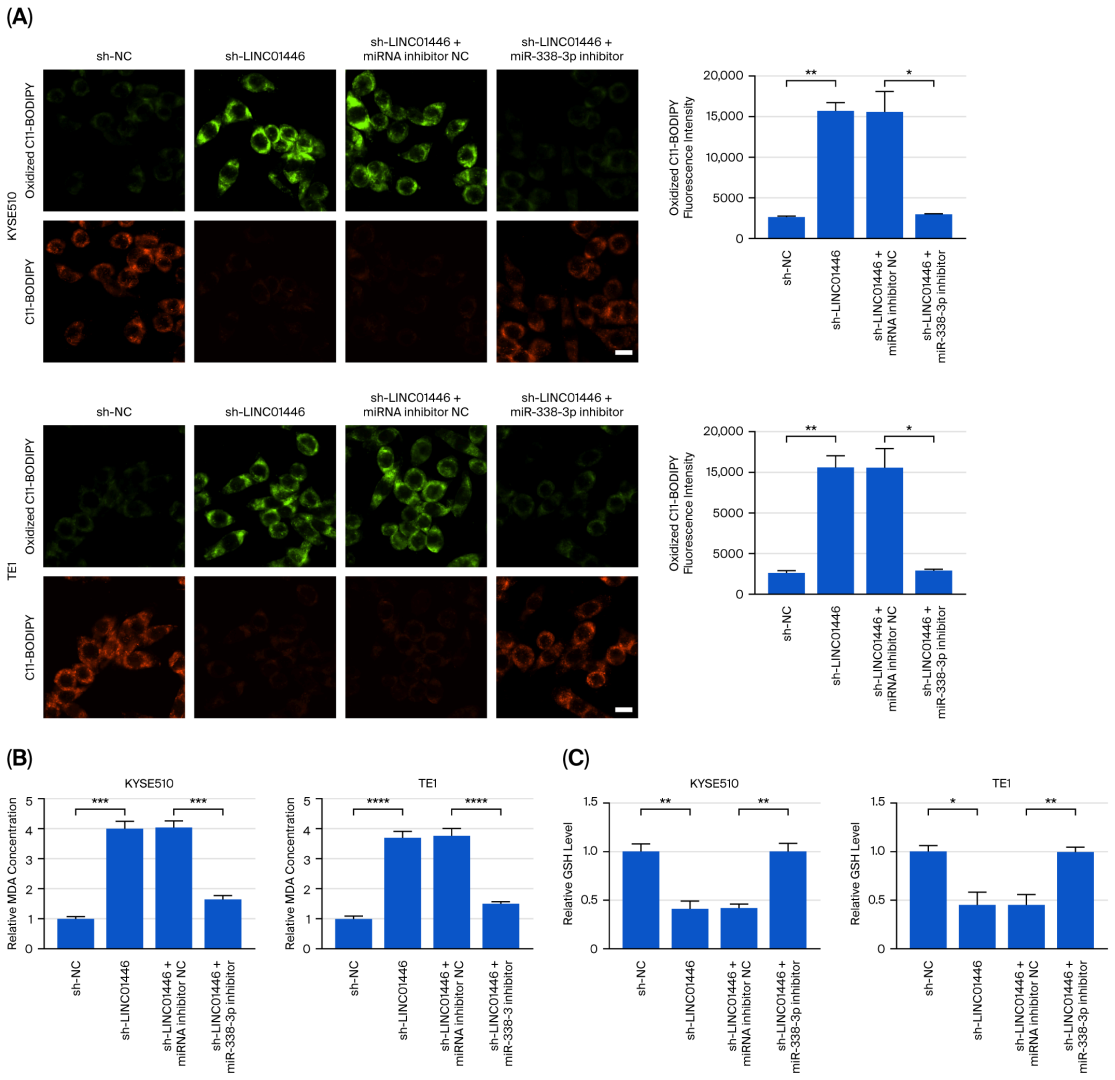
The effect of the LINC01446/miR-338-3p/APEX1 axis on ferroptosis in ESCC cells in vitro. (**A**) Effects of the LINC01446/miR-338-3p/APEX1 axis on intracellular lipid peroxidation levels in KYSE510 and TE1 cells, as detected using the C11-BODIPY probe (*n* = 3 biological replicates). The oxidized C11-BODIPY fluorescence intensity was analyzed using Student’s *t*-test. (**B**) Effects of the LINC01446/miR-338-3p/APEX1 axis on MDA levels in KYSE510 and TE1 cells (*n* = 3 biological replicates). MDA levels were analyzed using Student’s *t*-test. (**C**) The effect of the LINC01446/miR-338-3p/APEX1 axis on GSH levels in KYSE510 and TE1 cells (*n* = 3 biological replicates). GSH levels were analyzed using Student’s *t*-test. * *p* < 0.05, ** *p* < 0.01, *** *p* < 0.001, **** *p* < 0.0001.

**Figure 6:**
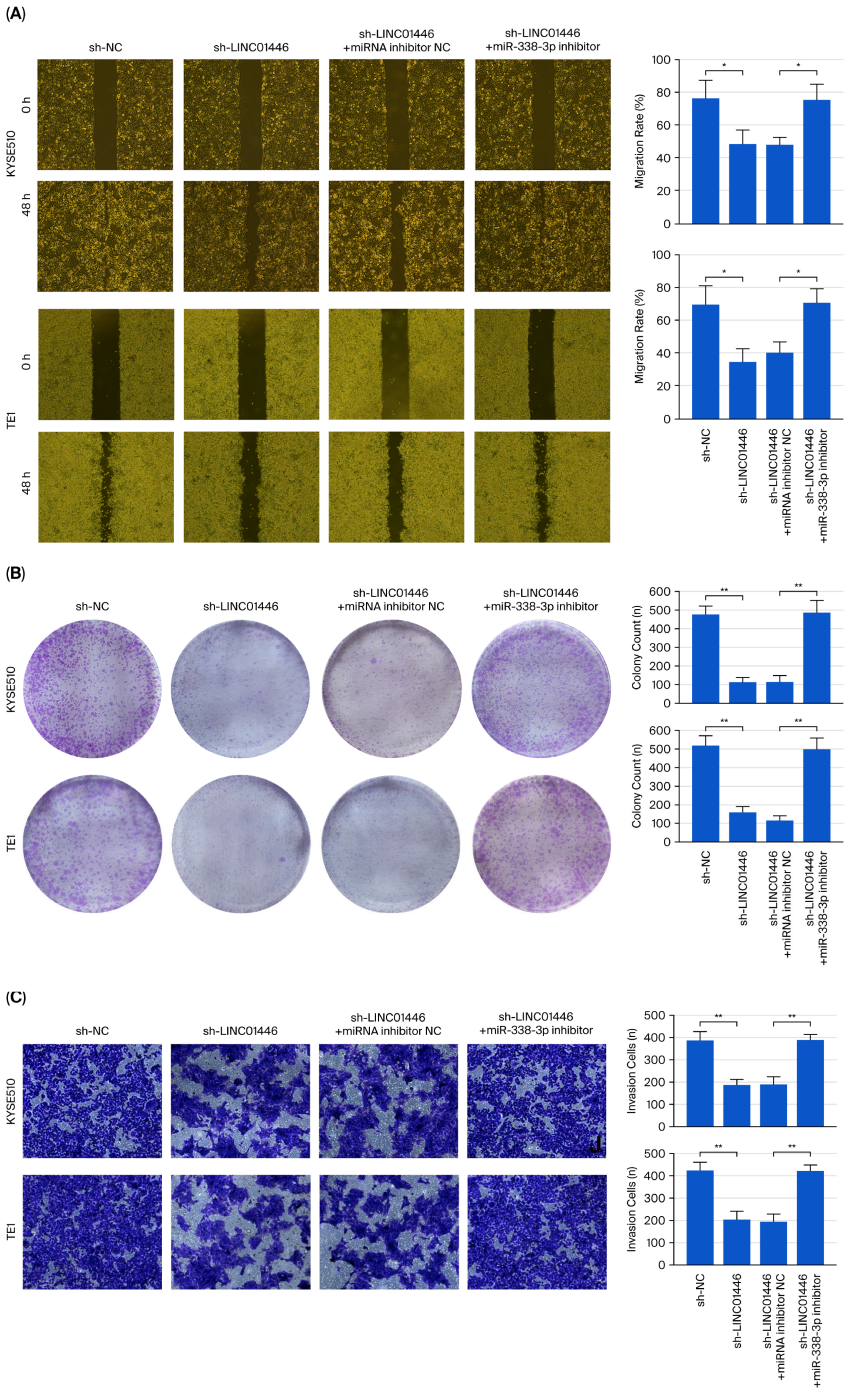
The effect of the LINC01446/miR-338-3p/APEX1 axis on the phenotypes of ESCC cells in vitro. (**A**) Effects of the LINC01446/miR-338-3p/APEX1 axis on the migratory ability of KYSE510 and TE1 cells, as evaluated by the wound healing assay (*n* = 3 biological replicates). The migration rates were analyzed using Student’s *t*-test. (**B**) Effects of the LINC01446/miR-338-3p/APEX1 axis on the proliferation capacity of KYSE510 and TE1 cells, as evaluated by the colony formation assay (*n* = 3 biological replicates). The counts of colonies were analyzed using Student’s *t*-test. (**C**) Effects of the LINC01446/miR-338-3p/APEX1 axis on the invasive ability of KYSE510 and TE1 cells, as evaluated by the Transwell invasion assay (*n* = 3 biological replicates). The numbers of invaded cells were analyzed using Student’s *t*-test. * *p* < 0.05, ** *p* < 0.01.

To verify the effect of the LINC01446/miR-338-3p/APEX1 axis on ferroptosis in ESCC, we conducted C11-BODIPY staining, MDA concentration quantification, and TEM. The results showed that LINC01446 knockdown significantly increased oxidized C11-BODIPY staining, elevated MDA concentrations, and decreased GSH levels in KYSE510 and TE1 cells, while co-transfection with the miR-338-3p inhibitor neutralized these alterations (Figure 5A–C). In addition, we performed colony formation, wound healing, and Transwell assays to determine the effect of the LINC01446/miR-338-3p/APEX1 axis on the phenotypes of ESCC cells. We found that LINC01446 knockdown significantly retarded colony formation, migration, and invasion in KYSE510 and TE1 cells (Figure 6A–C). In summary, these results demonstrate that the LINC01446/miR-338-3p/APEX1 axis contributes to ferroptosis defense and progression in ESCC cells in vitro.

**Figure 7:**
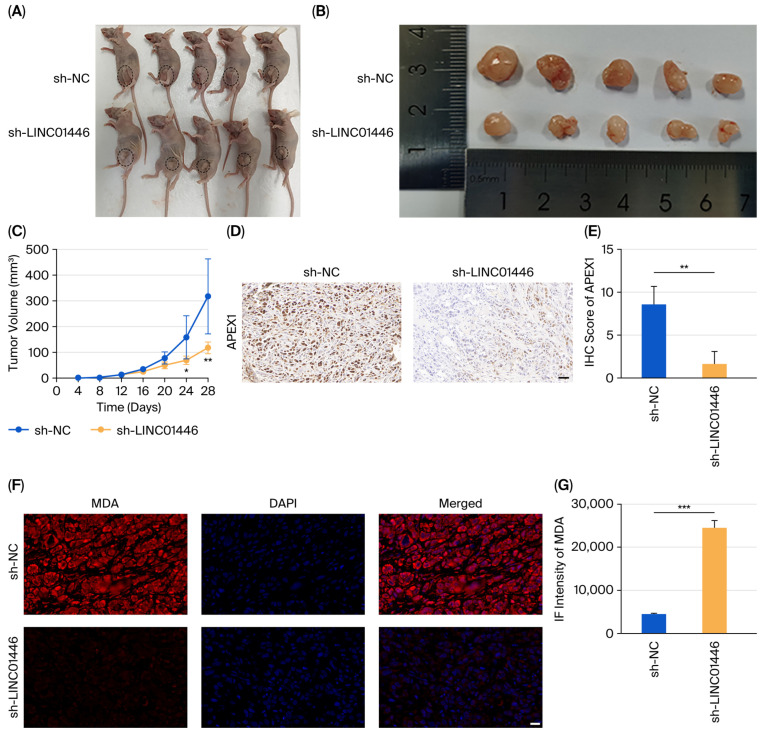
The effect of the LINC01446/miR-338-3p/APEX1 axis on the malignant behaviors and ferroptosis in ESCC cells in vivo. (**A**) Photograph of tumor-bearing mice constructed using KYSE510 cells after euthanasia. Subcutaneous tumor formation is indicated in the images. (**B**) Representative images of tumors formed of KYSE510 cells transfected with sh-LINC01446 or sh-NC. (**C**) Tumor volume growth curves of KYSE510 cells transfected with sh-LINC01446 or sh-NC (*n* = 5). The tumor volumes at the same time points were analyzed using Student’s *t*-test. (**D**) APEX1 expression in tumors formed of KYSE510 cells transfected with sh-LINC01446 or sh-NC, as detected by IHC. Scale bar = 50 μm. (**E**) Quantitative analysis of APEX1 expression levels in tumors formed of KYSE510 cells transfected with sh-LINC01446 or sh-NC (*n* = 5). The IHC scores were analyzed using Student’s *t*-test. (**F**) Representative images of MDA levels in tumors formed of KYSE510 cells transfected with sh-LINC01446 or sh-NC, as detected by immunofluorescence. Scale bar = 20 μm. (**G**) Quantitative analysis of MDA immunofluorescence intensity in tumors formed of KYSE510 cells transfected with sh-LINC01446 or sh-NC (*n* = 5). The MDA levels were analyzed using Student’s *t*-test. * *p* < 0.05, ** *p* < 0.01, *** *p* < 0.001.

Given that LINC01446 knockdown was associated with reduced migration and invasion and increased ferroptosis-related changes in ESCC cells in vitro, we established a xenograft mouse model to investigate the effect of LINC01446 knockdown on tumor growth in vivo. We found that LINC01446 knockdown significantly inhibited the growth of KYSE510-derived xenograft tumors (Figure 7A–C). To assess whether APEX1 expression was altered following LINC01446 knockdown in vivo, APEX1 expression in xenograft tissues was evaluated using IHC. APEX1 staining was mainly localized in the nuclei of tumor cells (Figure 7D). Notably, tumors from the sh-LINC01446 group showed significantly lower APEX1 IHC scores than those from the sh-NC group (1.60 ± 1.36 vs. 8.60 ± 1.96, *p* < 0.001, Figure 7E). Furthermore, we measured MDA levels to determine the effect of the LINC01446/APEX1 axis on lipid peroxidation in vivo, an accompanying phenomenon indicating the progression of ferroptosis. MDA levels were significantly higher in tumors derived from ESCC cells of the sh-LINC01446 group than in those of the sh-NC group (Figure 7F,G). Collectively, these in vivo findings suggest that LINC01446 knockdown suppresses ESCC xenograft growth and is accompanied by reduced APEX1 expression and increased ferroptosis.

## 4. Discussion

In the present study, we identified LINC01446 as an ESCC-associated lncRNA whose elevated expression was correlated with adverse clinicopathological features and poorer OS. Functional experiments showed that LINC01446 knockdown was associated with impaired proliferation, migration, and invasion, and increased ferroptosis-related changes in ESCC cells. Mechanistically, our data suggest that cytoplasmic LINC01446 may regulate APEX1 expression through interaction with miR-338-3p. Consistently, LINC01446 knockdown reduced xenograft growth in vivo and was accompanied by decreased APEX1 expression and elevated MDA levels in tumor tissues. These findings expand our current understanding of lncRNA-mediated regulatory networks in ESCC and suggest a potential link between LINC01446 dysregulation, APEX1-associated oxidative stress adaptation, and ferroptosis susceptibility.

A substantial body of research indicates that aberrant expression of lncRNAs plays a pivotal role in the pathogenesis and progression of various diseases, including cancer [16]. For instance, DANCR acts as a ceRNA to sponge and upregulate DDIT3, thereby promoting ESCC progression [18]. LINC00330 inhibits ESCC progression by disrupting the CCL2/CCR2 signaling axis and impeding CCL2-mediated reprogramming of tumor-associated macrophages [19]. Notably, certain lncRNAs can serve as biomarkers for predicting the prognosis of patients with tumors. For example, upregulated NEAT1 expression predicts poor progression-free survival (PFS) in EGFR-mutated lung adenocarcinoma patients receiving gefitinib as a first-line treatment [11]. In addition, TENM3-AS1 has shown great potential as a biomarker for predicting poor PFS and OS in gastric cancer (GC) [20]. Therefore, in this study, we focused on lncRNAs to identify novel diagnostic markers for ESCC.

By overlapping the bioinformatic analysis results from the TCGA ESCC cohort and GSE53625, we focused on LINC01446 to further explore its expression and role in ESCC. LINC01446 has been confirmed as a pro-oncogenic lncRNA in several cancers. For instance, in hepatocellular carcinoma (HCC), LINC01446 activates the SRPK2/SRSF1/VEGF_165_ axis to promote progression and angiogenesis [21]. Moreover, LINC01446 recruits LSD1 to the RASD1 promoter to suppress its transcription, thereby facilitating the proliferation and metastasis of GC cells [22]. In glioblastoma, LINC01446 promotes tumor cell proliferation and invasion by regulating the miR-489-3p/TPT1 axis [23]. Although multiple studies have revealed the significance of LINC01446 in certain cancer types, its expression and biological functions in ESCC remain elusive. We found that LINC01446 was highly expressed in ESCC tissues compared to that in normal esophageal tissues. Furthermore, we found that high LINC01446 expression was positively correlated with deeper tumor infiltration, positive lymph node metastasis, distant metastasis, and higher TNM staging in patients with ESCC. In addition, we found that high LINC01446 expression was negatively correlated with OS in ESCC patients, suggesting its potential as a prognostic biomarker.

We further explored the target genes of LINC01446 to demonstrate its oncogenic potential. By conducting RNA sequencing, we screened APEX1 as a candidate downstream gene of LINC01446 in ESCC. APEX1 is not only a key enzyme in the base excision repair pathway, where it processes apurinic/apyrimidinic sites generated during oxidative DNA damage, but also a redox regulatory protein that modulates the DNA-binding activity of several transcription factors, including AP-1, NF-κB, HIF-1α, and p53 [24]. These functions endow APEX1 with the potential to function as a pro-oncogenic protein, which may promote tumor phenotypes, such as proliferation and invasion. For instance, APEX1 promotes proliferation and metastasis in GC, NSCLC, melanoma, colorectal cancer, HCC, ovarian cancer, and gallbladder cancer [13,25,26,27,28,29,30]. In addition, APEX1 can promote angiogenesis by modulating the expression of various cytokines, such as FGF2, TGF-β, and VEGF [31,32,33]. In this study, we found that LINC01446 knockdown significantly reduced APEX1 expression, followed by a significant decrease in the proliferation, migration, and invasion capacities of ESCC cells.

The association between APEX1 and ferroptosis should be interpreted in the context of its dual functions in oxidative-stress responses. Ferroptosis is primarily driven by the iron-dependent accumulation of lipid peroxides and is critically opposed by the GSH/GPX4 antioxidant system [34]. Although APEX1 is not a canonical lipid peroxide-detoxifying enzyme such as GPX4, its DNA repair and redox-regulatory activities may increase the ability of cancer cells to tolerate reactive oxygen species and oxidative damage, thereby creating a cellular environment that is less permissive for ferroptosis. Thus, some researchers have hypothesized that APEX1 may exert an inhibitory effect on ferroptosis [12]. Subsequent studies have preliminarily confirmed the role of APEX1 in ferroptosis defense [35,36,37,38]. In ESCC, our findings both corroborate and extend previous observations: LINC01446 knockdown reduced APEX1 expression and induced ferroptosis-associated changes, including increased lipid peroxidation and MDA levels and decreased GSH levels; importantly, these effects were attenuated by Fer-1. These findings suggest that LINC01446 may promote an aggressive phenotype and ferroptosis defense in ESCC cells by regulating APEX1 expression.

Furthermore, APEX1 has been demonstrated to be a biomarker associated with poor prognosis in various malignant tumors, including testicular cancer, HCC, GC, osteosarcoma, head and neck cancer, and colon cancer [29,39,40,41,42,43]. However, its expression and prognostic significance in ESCC remain unclear. Therefore, we determined its expression in ESCC and analyzed its correlation with the OS of the patients. The results demonstrated that APEX1 was significantly upregulated in ESCC compared to that in normal esophageal tissue. More importantly, we found that high APEX1 expression was inversely correlated with OS in ESCC patients and might serve as a biomarker for predicting prognosis.

Our study extends previous evidence by identifying an upstream noncoding RNA-dependent regulatory mechanism of APEX1 in ESCC. Specifically, LINC01446 acts as a molecular sponge for miR-338-3p and relieves miR-338-3p-mediated repression of APEX1, which may enhance the antioxidant and stress-adaptive capacities of ESCC cells. First, we predicted the subcellular localization of LINC01446 using the lncLocator online tool. We found that the primary subcellular localization of LINC01446 was cytoplasmic, which was subsequently confirmed by FISH and nuclear and cytoplasmic fraction isolation experiments. One of the primary mechanisms through which cytoplasm-localized lncRNAs exert their biological functions is by acting as ceRNAs to sponge miRNAs, thereby regulating the target mRNAs [44]. Although this mechanism of action for lncRNAs was initially proposed as a hypothesis, subsequent studies have increasingly provided evidence for this mechanism [8,11,18,45,46]. In this study, we predicted that LINC01446 might upregulate APEX1 expression by sponging miR-338-3p using an online bioinformatics database, and this result was further validated using a dual-luciferase reporter assay, AGO2-RIP, and miRNA rescue experiments. miR-338-3p is an miRNA that has been shown to have tumor-suppressive effects in various malignant tumors, including breast cancer, colon cancer, and pancreatic cancer [11,47,48,49,50]. In ESCC, miR-338-3p has been demonstrated to exert an anticancer effect by regulating the expression of proteins such as SOX4, IGF1R, and MAP3K9 [51,52,53]. Moreover, we found that the LINC01446/miR-338-3p/APEX1 axis might promote progression and ferroptosis defense in ESCC cells through in vitro and in vivo experiments. Thus, the mechanistic insights gained from this study reinforce the importance of the LINC01446/miR-338-3p/APEX1 axis in ESCC.

From a translational perspective, the LINC01446/miR-338-3p/APEX1 axis may represent a potential biomarker-guided therapeutic vulnerability in ESCC. The concurrent pattern of high LINC01446 and APEX1 expression, together with reduced miR-338-3p activity, may help identify a subset of tumors with enhanced oxidative-stress tolerance and a more aggressive clinical phenotype. Therapeutically, LINC01446 knockdown by antisense oligonucleotides or small interfering RNAs, as well as restoration of miR-338-3p activity using miRNA mimics, could potentially reduce APEX1 expression and impair some malignant behaviors of ESCC cells. However, RNA-based therapeutic approaches are increasingly being explored in cancer, although efficient tumor-selective delivery, in vivo stability, and potential off-target effects remain important challenges. In parallel, several small-molecule inhibitors targeting APEX1 are being investigated in various malignant tumors (such as esophageal adenocarcinoma and pancreatic cancer) and have demonstrated promising results in preclinical studies [54,55]. Our study provides a theoretical foundation for investigating the potential role and translational applications of APEX1-targeted therapies in ESCC. Additionally, future studies using multiple ESCC models, patient-derived organoids or xenografts, and in vivo delivery systems will be necessary to determine whether LINC01446 expression, miR-338-3p status, and APEX1 expression can be used for patient stratification and to assess the clinical feasibility of targeting this axis.

However, this study has several limitations. First, the regulatory network involving additional miRNAs and the downstream target genes of LINC01446 remain to be elucidated. Second, the approach used to detect ferroptosis had certain methodological limitations; therefore, the role of ferroptosis resistance mediated by the LINC01446/miR-338-3p/APEX1 axis in ESCC progression requires further investigation. Third, our mechanistic analysis of LINC01446 in ESCC focused primarily on the miR-338-3p/APEX1 pathway and did not examine other potentially relevant mechanisms. Consequently, the precise biological functions of LINC01446 in ESCC remain incompletely understood. Fourth, only one ESCC cell line was used to establish the xenograft mouse model, which may limit the generalizability of our findings. Finally, the study included an insufficient number of clinical samples for external validation, and no therapeutic interventions were evaluated in the animal experiments. Thus, the evidence supporting the clinical significance of our findings should be interpreted with caution.

## 5. Conclusions

In summary, our findings indicate that LINC01446 is upregulated in ESCC and is associated with unfavorable clinicopathological characteristics and poor OS. Bioinformatic analyses, functional experiments, and mechanistic investigations suggest that LINC01446 may modulate APEX1 expression by competitively interacting with miR-338-3p. This regulatory axis is associated with enhanced malignant phenotypes and reduced ferroptosis in ESCC. Collectively, these results support a potential role for the LINC01446/miR-338-3p/APEX1 axis in ESCC progression and suggest that LINC01446 and APEX1 may represent candidate prognostic biomarkers and therapeutic targets for further investigation.

## Data Availability

Data in this study will be made available from the corresponding author upon reasonable request. The dataset used in this study is available from the following source: GEO dataset (GSE53625, https://www.ncbi.nlm.nih.gov/geo/query/acc.cgi?acc=GSE53625, (accessed on 31 August 2020)).

## Supplementary Materials

The following supporting information can be downloaded at: https://www.mdpi.com/article/10.3390/cancers18152496/s1, Figure S1: Heatmap showing the differentially expressed lncRNAs between ESCC and normal esophageal tissues in the TCGA database; Figure S2: Heatmap showing the results of differential lncRNA analysis between ESCC and normal esophageal tissues in the GSE53625 dataset; Figure S3: Correlation of LINC00392, LINC00942, LINC01614, LINC00460, LINC00707, LINC01615, POU6F2-AS2, LINC00626, and LINC01592 with OS in ESCC patients; Figure S4: The effect of LINC01446 on phenotypes and ferroptosis in ESCC cells in vitro; Table S1: Ferroptosis-related genes downloaded from Ferrdb v2; Table S2: Primer sequences for qRT-PCR; Table S3: The sequences of shRNAs for knocking down LINC01446.

## Abbreviations

The following abbreviations are used in this manuscript:

ESCC	Esophageal squamous cell carcinoma
lncRNA	Long non-coding RNA
OS	Overall survival
MDA	Malondialdehyde
APEX1	Apurinic/apyrimidinic endodeoxyribonuclease 1
ceRNA	Competing endogenous RNA
UTR	Untranslated region
miRNA	MicroRNA
RIP	RNA immunoprecipitation
FISH	Fluorescence in situ hybridization
shRNA	Short hairpin RNA
GSH	Glutathione
MTDH	Metadherin
GC	Gastric cancer
HCC	Hepatocellular carcinoma
