# Supplementary material for: LINC01446/miR-338-3p/APEX1 Axis Promotes Ferroptosis Defense and Progression in Esophageal Squamous Cell Carcinoma
Source: Cancers (Basel). 2026 Aug 4;18(15):2496. doi: 10.3390/cancers18152496 (PMC13464326; doi:10.3390/cancers18152496)
Supplement: Supplementary file 1 [file cancers-18-02496-s001.zip › Figure legends for supplemetary figures.pdf]

Figure S1.

Heatmap showing the differentially expressed lncRNAs between ESCC and normal esophageal tissues in the TCGA database.

Figure S2.

Heatmap showing the results of differential lncRNA analysis between ESCC and normal esophageal tissues in the GSE53625 dataset.

Figure S3.

Correlation of LINC00392, LINC00942, LINC01614, LINC00460, LINC00707, LINC01615, POU6F2-AS2, LINC00626, and LINC01592 with OS in ESCC patients.

Figure S4.

The effect of LINC01446 on phenotypes and ferroptosis in ESCC cells in vitro. (**A**) Effects of LINC01446 knockdown on intracellular lipid peroxidation levels in TE1 cells, as detected using the C11-BODIPY probe. (**B**) Quantitative analysis of MDA, a lipid peroxidation product, in TE1 cells after LINC01446 knockdown. (**C**) Effects of LINC01446 knockdown on GSH level in TE1 cells. (**D**) Effect of LINC01446 knockdown on the colony formation ability of KYSE510 cells. (**E**) Effect of LINC01446 on the migration of TE1 cells, as detected using the wound-healing assay. (F) Effect of LINC01446 on the invasion of KYSE510 cells, as detected using the transwell assay. *$p < 0.05$, **$p < 0.01$, ***$p < 0.001$, ****$p < 0.0001$.
