# Supplementary material for: LINC01446/miR-338-3p/APEX1 Axis Promotes Ferroptosis Defense and Progression in Esophageal Squamous Cell Carcinoma
Source: Cancers (Basel). 2026 Aug 4;18(15):2496. doi: 10.3390/cancers18152496 (PMC13464326; doi:10.3390/cancers18152496)
Supplement: Supplementary file 1 [file cancers-18-02496-s001.zip › Table S1.pdf]

Table S1 Ferroptosis-related genes downloaded from Ferrdb v2

Ferroptosis driver genes	Ferroptosis suppressor genes
ABCC1	ABCC5
ACADSB	ABHD12
ACO1	ACOT1
ACSF2	ACSL3
ACSL1	ADAMTS13
ACSL4	ADIPOQ
ACVR1B	AHCY
ADAM23	AIFM2
AEBP2	AKR1C1
AGPAT3	AKR1C2
AGPS	AKR1C3
ALOX12	AKT1S1
ALOX12B	ALDH3A2
ALOX15	AR
ALOX15B	ARF6
ALOX5	ARNTL
ALOXE3	ASAH2
AMN	ATF2
ANO6	ATF4
AQP3	BCAT2
AQP5	BEX1
AQP8	BRD2
ATF3	BRD3
ATF4	BRD4
ATG13	BRDT
ATG16L1	CA9
ATG3	CAMKK2
ATG4D	CAV1
ATG5	CBS
ATG7	CD44
ATM	CDC25A
ATP5MC3	CDH1
BACH1	CDKN1A
BAP1	CHMP1A
BECN1	CHMP5
BID	CHMP6
BRD7	CISD1
BRPF1	CISD2
CARS1	CISD3
CCDC6	COPZ1

CD82	CP
CDCA3	CREB1
CDKN2A	CREB3
CDO1	CREB5
CFL1	DAZAP1
CGAS	DECR1
CHAC1	DHODH
CHP1	ECH1
CIRBP	ENO3
CLTRN	ENPP2
COX4I2	ETV4
CPEB1	EZH2
CS	FABP4
CTSB	FADS2
CYB5R1	FANCD2
CYBB	Fer1HCH
CYGB	FGF21
CYP4F8	FH
DCAF7	FNDC5
DDR2	FTH1
DLD	FTL
DNAJB6	FTMT
DPEP1	FURIN
DPP4	FXN
DUOX1	FZD7
DUOX2	G6PD
EGFR	GALNT14
EGLN2	GCH1
EGR1	GCLC
ELAVL1	GDF15
ELOVL5	GLRX5
EMC2	GOT1
EPAS1	GPX4
EPT1	GSTM1
FADS1	HCAR1
FADS2	HELLS
FAR1	HIF1A
FBXW7	HMOX1
FLT3	HSF1
FOXO4	HSPA5
G6PD	HSPB1
G6PDX	IDH2
GABARAPL1	IL6

GABARAPL2	ISCU
GJA1	JUN
GLS2	KDM3B
GOT1	KDM4A
GPAT4	KIF20A
GRIA3	KLHDC3
GSK3B	LAMP2
GSTZ1	LCN2
HDDC3	MAPKAP1
HIF1A	MARCHF5
HILPDA	MEF2C
HMGB1	MGST1
HMOX1	MLST8
HRAS	MPC1
IDH1	MS4A15
IDO1	MT1G
IFNA1	MTF1
IFNA10	MTOR
IFNA13	MUC1
IFNA14	NCOA3
IFNA16	NEDD4
IFNA17	NEDD4L
IFNA2	NF2
IFNA21	NFE2L2
IFNA4	NFS1
IFNA5	NOS2
IFNA6	NQO1
IFNA7	NR4A1
IFNA8	NR5A2
IFNG	NT5DC2
IL1B	NUPR1
IL6	OTUB1
INTS2	P4HB
IREB2	PANX2
KDM5A	PARK7
KDM5C	PARP1
KDM6B	PARP10
KEAP1	PARP11
KLF2	PARP12
KMT2D	PARP14
KRAS	PARP15
LCE2C	PARP16
LGMN	PARP2

LIFR	PARP3
LIG3	PARP4
LONP1	PARP6
LPCAT3	PARP8
LPIN1	PARP9
LYRM1	PDK4
MAP1LC3A	PDSS2
MAP3K11	PEDS1
MAP3K14	PIK3CA
MAPK1	PIR
MAPK14	PLA2G6
MAPK3	PLIN2
MAPK8	PML
MAPK9	PPARA
MDM2	PPARD
MDM4	PPP1R13L
METTL14	PRDX1
MFN2	PRDX6
MIB1	PRKAA2
MIB2	PROK2
MICU1	PROM2
MIOX	PRR5
MLLT1	PTPN18
MMD	RARRES2
MT1DP	RB1
MTCH1	RBMS1
MTDH	RELA
MYB	RHEBP1
MYCN	RICTOR
NCOA4	RNF113A
NDRG1	RPTOR
NOX1	RRM2
NOX3	SCD
NOX4	SENP1
NOX5	SESN2
NR1D1	SIAH2
NR1D2	SIRT1
NRAS	SIRT2
OSBPL9	SIRT3
PANX1	SIRT6
PAQR3	SLC16A1
PEBP1	SLC3A2
PEX10	SLC40A1

PEX12	SLC7A11
PEX2	SMPD1
PEX3	SOX2
PEX6	SQSTM1
PGD	SRC
PGRMC1	SREBF1
PHF21A	SREBF2
PHKG2	SRSF9
PIEZO1	STAT3
PIK3CA	STK11
POM121L12	SUV39H1
POR	TERT
PPARG	TF
PRKAA1	TFAM
PRKAA2	TFAP2A
PRKCA	TFRC
PTEN	TMBIM4
PTPN6	TMSB4X
QSOX1	TMSB4Y
RPL8	TP53
SAT1	TP63
SCP2	TRIB2
SIRT1	TXN
SIRT3	TYRO3
SLC11A2	USP11
SLC1A5	USP35
SLC25A28	VCP
SLC38A1	VDAC2
SLC39A14	VDR
SLC39A7	ZFP36
SLC7A11	
SMAD7	
SMG9	
SMPD1	
SNCA	
SNX4	
SNX5	
SOCS1	

STING1	
TAFAZZIN	
TBK1	
TF	
TFR2	
TFRC	
TGFB1	
TGFBR1	
TIMM9	
TIMP1	
TLR4	
TNFAIP3	
TOR2A	
TP53	
TRIM21	
TRIM26	
TRIM46	
TSC1	
TTPA	
ULK1	
ULK2	
USP11	
USP7	
VDAC2	
WIPI1	
WIPI2	
WWTR1	
YAP1	
YTHDC2	

YY1AP1	
