# Supplementary material for: LINC01446/miR-338-3p/APEX1 Axis Promotes Ferroptosis Defense and Progression in Esophageal Squamous Cell Carcinoma
Source: Cancers (Basel). 2026 Aug 4;18(15):2496. doi: 10.3390/cancers18152496 (PMC13464326; doi:10.3390/cancers18152496)
Supplement: Supplementary file 1 [file cancers-18-02496-s001.zip › Table S2.pdf]

Table S2 Primer sequences for qRT-PCR

Name	Primer sequence (5'-3')
**LINC01446**	F: AGCCAGTTCCCCAAGTAAGC
	R: GGACTGCTTCATCTCTCCCG
**GAPDH**	F: ACAACTTTGGTATCGTGGAAGG
	R: GCCATCACGCCACAGTTTC
**miR-338-3p**	RT: GTCGTATCCAGTGCGTGTCGTGGAGTCGGCAATTGCACTGGATACGACCAACAAA
	F: ACAACTTTGGTATCGTGGAAGG
	R: TGCTTCCAGCATCAGTGAT
**U6**	RT: CGCTTCACGAATTTGCGTGTCAT
	F: GCTTCGGCAGCACATATACTAAAAT
	R: CGCTTCACGAATTTGCGTGTCAT
