# Supplementary material for: LINC01446/miR-338-3p/APEX1 Axis Promotes Ferroptosis Defense and Progression in Esophageal Squamous Cell Carcinoma
Source: Cancers (Basel). 2026 Aug 4;18(15):2496. doi: 10.3390/cancers18152496 (PMC13464326; doi:10.3390/cancers18152496)
Supplement: Supplementary file 1 [file cancers-18-02496-s001.zip › Table S3.pdf]

Table S3 The sequences of shRNAs for knocking down LINC01446

Name	Sequence (5'-3')
Sense	GAGCAGTTGTTGAAGATGATTCAAGAGATCATCTTCAACAACTGCTCCTTTTTTG
Anti-sense	AATTCAAAAAAGAGCAGTTGTTGAAGATGATCTCTTGAATCATCTTCAACAACTGCTC
